# Herd-level risk factors for chronic pleurisy in finishing pigs: a case-control study

**DOI:** 10.1186/s40813-020-00156-0

**Published:** 2020-09-02

**Authors:** Outi Hälli, Minna Haimi-Hakala, Claudio Oliviero, Mari Heinonen

**Affiliations:** Department of Production Animal Medicine, University of Helsinki, Paroninkuja 20, FI-04920 Saarentaus, Finland

**Keywords:** Pleurisy, *Actinobacillus pleuropneumoniae*, Swine influenza virus, Herd type, Meat inspection, Respiratory disease complex

## Abstract

**Background:**

Chronic pleurisy is a common finding in slaughtered pigs in post-mortem meat inspection. The prevalence of pleurisy has been increasing during the last decade also in Finland. The aim of this prospective case-control study was to search for environmental, infectious and management-related herd-level risk factors for pleurisy in the slaughterhouse. Altogether 46 Finnish pig herds, including 25 control (low pleurisy prevalence in meat inspection) and 21 case (high pleurisy) herds, were enrolled in the study and visited during the tenth week of the rearing period of finishing pigs. Herd personnel were asked about basic herd information, management and environmental factors. Selected pigs were examined clinically, environmental parameters were measured and 15 blood samples per herd were taken during herd visits. Antibodies against *Actinobacillus pleuropneumonia* serotype 2 (APP2) and ApxIV toxin and swine influenza virus were measured. After the slaughter of study pigs, meat inspection results of the batch were gathered from slaughterhouses. Multivariate logistic regression model was built to identify possible risk factors for a herd to be a case herd (i.e. having high pleurisy values).

**Results:**

Finishing herd type and herd size were observed to act as risk factors. None of clinical signs of pigs, management-related factors or environmental measurements were associated with herd status.

**Conclusions:**

As previously known, in endemic and subclinical infections such as APP, herd factors are important, but detailed risk factors seem to be difficult to identify.

## Background

Chronic pleurisy is a common finding in slaughtered pigs in post-mortem meat inspection. Pleurisy prevalence around 20% has been detected in several European countries [1–4]. In Finland, pleurisy prevalence in slaughter pigs has clearly been increasing during the last decade [5]. The registration of pleurisy has continuously increased also in a neighbouring country of Finland, Sweden, since the year 2000 [6].

Most studies investigating pleurisy have been done in countries with many possible pathogens present in the pig population. In Finland, the prevalence of porcine respiratory pathogens differs from the situation in continental Europe. The country has been free from Aujeszky disease virus (ADV), porcine respiratory corona virus (PRCV) and porcine reproductive and respiratory syndrome virus (PRRSV) for decades and nearly free from *Mycoplasma hyopneumoniae* (MHyo). Porcine respiratory disease complex is a multifactorial syndrome with clinical signs caused usually by multiple micro-organisms, both bacteria, (e.g. *Actinobacillus pleuropneumoniae (*APP*), Pasteurella multocida* and *Glaesserella parasuis)*, and viruses together with environmental and management-related factors as well as genetics. Both viruses and MHyo have been considered as primary pathogens, which predispose pigs to concomitant bacterial infections such as APP [7]. Seropositivity to MHyo was reported to increase the odds of contracting chronic pleurisy [8], and APP serotype 2 together with PRRSV was described to be significantly associated with pleurisy [9]. Also, swine influenza virus (SIV) has been noted to have an association with pleurisy [10]. Especially dorsocaudal pleurisy has been reported to be strongly associated with APP [1, 6, 10, 11].

The type and severity of the disease caused by APP is considered to depend on factors related to pathogen virulence, host, environment and management [12]. Jirawattanapong et al. [13] found no single cause of pleurisy in their study in the Netherlands investigating possible infectious causes. They suggested the combined cause to be a variety of infectious agents together with environmental factors. While experimental studies have helped us to gather information about the pathogens, knowledge of the interplay of different factors affecting disease outcome in commercial pig farms remains incomplete [12]. Hence, the swine producing community lacks practical and effective means for pleurisy control. The aim of this epidemiological case-control study in commercial swine herds was to search for various environmental, management-related and infectious factors increasing risk for high pleurisy in slaughtered pigs. If the main risk factors at herd level were known, we could target control measures more effectively.

## Methods

This study was case-control study with a herd as the unit of interest. A case-control study design was selected to find a clear difference in an outcome that was originally measured in a continuous scale (percentage of pleurisy in study farms). The target population included medium- to large-sized (more than 500 pigs per herd) Finnish herds rearing finishing pigs.

### Sampling and data gathering

Purposive sampling was used. A study herd needed to fulfill the inclusion criteria of 1) at least 1000 finishing pigs sent for slaughter annually and 2) location in south-western Finland within a distance of 250 km from the University of Helsinki ambulatory clinic in Mäntsälä. Three major slaughterhouses were asked to compile a list of finishing pig herds fulfilling the above criteria. This list including 219 herds served as a sampling frame. At the same time, the slaughterhouses provided the research group with the pleurisy percentage of these herds during the year preceding enrolment in the study. The herds were then sorted in descending order according to their pleurisy percentage, separately for each slaughterhouse. Herds were tentatively divided into case and control herds based on their pleurisy values. Herds having a higher pleurisy value than slaughterhouse-specific mean pleurisy plus standard deviation value were considered to be tentative case herds. Similarly, herds having a lower pleurisy value than the slaughterhouse mean minus the standard deviation were considered to be tentative control herds. For slaughterhouse one, pleurisy percentage (±standard deviation) used in tentative classification was 1.9 ± 1.4%, for slaughterhouse two 27.5 ± 16.8% and for slaughterhouse three 4.0 ± 2.9%. These tentative herd classifications were verified based on the post mortem findings of the study batch as described later in materials and methods.

Researchers then contacted the tentative case and control herds and requested their consent to participate in the study. During the first contact researchers ascertained that the herd had not suffered from acute respiratory disease outbreak during the last batch of finishing pigs. All voluntary herds were enrolled in the study until the desired number of herds (at least 60 herds representing all three slaughterhouses proportionally to their market share) was fulfilled. Of 93 herds contacted, 29 (31.2%) opted out or were unable to participate. Out of herds that opted out, 48% were not willing to participate, 48% no longer had animals or had changed slaughterhouse, making participation impossible, and 3% reported some other reason. Customers of slaughterhouse 1 and case herds (54% cases vs. 45% controls) were overrepresented in herds opting out. Opted-out herds had approximately twice as many animals as herds that were willing to participate.

Researchers visited all study herds once about 10 weeks after the growers (body weight 25 kg) had arrived at the finishing pig herd or a finisher compartment in a farrow-to-finish herd. Visits were carried out during years 2012–2014. At the beginning of the visit, the researchers ascertained that the pig groups had not suffered from acute respiratory disease outbreak after entering the finishing herd or compartment. The herd owner or responsible caretaker was interviewed about basic information of the herd (herd type, number of animals), management and environmental factors using a herd visit questionnaire. The main categories of management-related information included vaccination routines, biosecurity and hygiene, animal flow, mean number of animals per compartment and per pen, mean size of pen, medication routines and feeding. The main categories of environmental factors were ventilation, temperature, heating and floor type.

In each herd, one finishing pig compartment containing at least 200 but less than 1000 finishing pigs was chosen for clinical inspection of pigs and environmental measurements. After carefully entering the compartment, the researchers counted the number of pens containing pigs lying on top of each other. After that, pen wall temperature (Testo 830-T1, Testo SE & Co, Germany), NH_3_ concentration (Dräger-Tube Pump Accuro, Drägerwerk AG & Co, Germany) and air temperature, humidity and airflow (Envic DM-101, Envic Oy, Finland) were measured in four different spots (two opposite corner pens and two pens in the middle of the row) of the compartment. A herd-level mean of these four measurements was used in further analyses. After environmental measurements, all pigs were gently forced to stand up and their coughing and sneezing episodes were counted for 5 min. A coughing/sneezing episode was defined as a single cough/sneezing or as a set of continuous coughs/sneezings of one animal. The number of soiled pens and the presence of various clinical signs (tear staining, conjunctivitis, neurological signs, lameness, acute and healed tail biting, diarrhoea, flank biting or skin scratches, runts and sitting pigs) were registered individually in approximately 100 pigs.

Altogether 15 pigs were selected evenly in different parts of the study compartment for blood sampling. These pigs were caught with a snout snare and a blood sample was taken from their vena jugularis with vacuum needles and serum tubes. The samples were centrifuged the next day in the laboratory, and the serum was stored at − 20 °C until analysed.

At the end of the visit, outdoor temperature was measured. Before leaving the herd, herd personnel were asked to inform researchers about any major unexpected events, e.g. acute disease outbreak or equipment malfunction, during the remaining rearing period.

After the study batch containing clinically examined animals was sent to slaughter, the slaughterhouses provided the researchers with the meat inspection (MI) findings of the batch, including mean carcass weight, kilograms of condemned meat, percentage of whole and partial carcass condemnations, lean meat percentage, abscesses, arthritis, milk spots in liver, organ condemnations, tail biting, pneumonia and pleurisy. Herds having pleurisy percentage higher than the slaughterhouse-specific mean pleurisy in the batches undergoing analysis were considered batch-specific case herds. Similarly, herds having pleurisy percentage lower than the slaughterhouse mean were considered batch-specific control herds. These batch-specific results were used to verify the herd classification. Batch-specific mean pleurisy percentages used for classification were 1.6, 30.2 and 1.8% for slaughterhouses 1, 2 and 3, respectively. Finally, the group of confirmed case herds included only tentative case herds that were also batch-specific case herds. Similarly, confirmed control herds included only those tentative control herds that were also batch-specific control herds.

### Laboratory analysis

APP antibodies were measured using two commercial test kits: IDEXX APP-ApxIV ELISA (IDEXX, Liebefeld-Bern, Swizerland) to detect antibodies against ApxIV toxin, which is produced by all known APP serotypes (19), and IDvet ID Screen APP 2 indirect ELISA (IDvet, Grabels, France) to detect antibodies against lipopolysaccharides (LPS) specific to APP serotype 2 (APP2), with a sensitivity of 82.9% and a specificity of 99.6% for IDEXX APP ApxIV ELISA and a specificity of 99.68% for IDVet APP2 ELISA. A pig was considered positive if the test used detected any antibodies in the serum sample.

All blood samples were tested with influenza A antibody ELISA (ID Screen® Influenza A Antibody Competition, IdVet, Grabels, France) according to the manufacturer’s instructions. A sample was considered unclear when the competition percentage (S/N%) was 46–49% and positive when the competition percentage was ≤45%. If a herd had at least one unclear or positive blood sample (pig) in the ELISA test, blood samples of that herd were further analysed using a haemagglutination inhibition (HI) test according to the European Surveillance Network for Influenza in Pigs. This was done with the antigens H1N1 (SW/Best/96), H1N2 (SW/Gent/7625/99) and H3N2 (SW/St. Oedenrode/96). All antigens were provided by GD Animal Health Service (Deventer, the Netherlands). A sample was considered HI positive if the HI titre was ≥1:20.

### Statistical analysis

A required sample size of 24 herds in both groups (control and case) was calculated assuming the proportion of exposed as 40% for controls and as 80% for cases in presumably the most influential variable (herd type). Alpha 0.05 and power 0.8 were used. The least extreme odds ratio to be detected is 6.0 [14].

All gathered data were scrutinized, and all unreliable answers were either checked and corrected, or if this was impossible, removed from the dataset. Most of the variables describing management-related and environmental factors were transformed into meaningful categories. Most of the count variables and measurements were handled as continuous variables.

The outcome variable was a categorical variable “confirmed case or control”. Regarding the predictors, the herd-level prevalence (%) of different clinical signs was calculated. Furthermore, the percentage of soiled pens or pens where pigs were lying on top of each other was calculated. The prevalence of diarrhoea, neurological signs or skin scratches and soiled pens was almost zero and these variables were not included in further analyses. For modelling serological results, herd-level APP2, APPIV and SIV antibody prevalences (%) were calculated. Both APP2 and APPIV seroprevalences were categorized in two categories (low/high prevalence within herd, median used as a cut-off point). Descriptive statistics of all predictor variables at herd level (management-related factors, clinical signs, environmental measurements, and serology) were compiled containing all herds, and a comparison between case and control herds was carried out.

Univariate associations between predictor variables and outcome were evaluated using logistic regression. The liberal p-value of 0.2 was used as a keep-in or drop-out threshold. The correlations between predictor values were scrutinized. The herd type (farrow-to-finish or finishing only) was detected to correlate strongly with many predictor variables (e.g. room-level all-in all-out production). The decision to force the variable “herd type” in further models and drop correlating, intervening variables was made. None of the variables related to environmental measurements showed to be statistically significant and therefore were not included in the multivariable model. Furthermore, SIV serology univariate association with herd pleurisy status was clearly insignificant and this variable was not included in the further modeling.

Finally, a multivariable logistic regression model was built. The initial model contained predictor variables: herd type (fattening or farrow-to-finish), number of finishing pigs in the herd, compartment disinfection (always between batches, sometimes, never), littering frequency (once or twice per day or continuously available), proportion of slatted flooring (≤50%/> 50%), airspace per pig, feeding type (liquid/dry), piggery temperature when weaners entered the compartment, piggery temperature when finishing pigs left the compartment, heating (yes/no), ventilation system service (yes/no), ventilation adjustment difference in winter, loading corridor (yes/no), handwashing facility for visitors (yes/no), sitting pigs (%), flank biting (%), conjunctivitis (%), herd-level APP2 prevalence and APPIV antibody prevalence (high/low). Backward elimination model building strategy was then utilized. The final model contained only the predictor variables herd type and number of finishing pigs per herd.

For model diagnostics, the basic assumptions of logistic models were inspected with regard to data structure and nature of the predictor variables. Observations were independent from each other and the continuous variable in the final model (no. of finishing pigs) had a sufficiently linear relationship with the outcome. The Hosmer-Lemeshow goodness of fit test with seven groups collapsed on quantiles of estimated probabilities was used, and it gave a non-significant result (*p* = 0.5) as the null hypothesis is “model fits the data”. In addition, residuals, deltabeta and leverage values were scrutinized to observe potential outliers. Furthermore, the area under the ROC curve (0.92) was evaluated to assess predictive ability of model.

## Results

Originally, data were available from 64 herds (28 tentative case and 36 tentative control herds). We removed 18 of the tentative herds because they did not have the same pleurisy status in the batch inspected after the herd visit. Therefore, 46 study herds (21% of eligible herds in sampling frame and 49% of eligible herds contacted) remained, 25 control and 21 case, for data analysis. These herds belonged in three different slaughterhouses (slaughterhouse 1, 27 herds, 59%; slaughterhouse 2, 14 herds, 30%; slaughterhouse 3, 5 herds, 11%). A slight majority of herds reared only finishing pigs (26 herds, 57%) and the rest were farrow-to-finish herds (20 herds, 43%).

Basic descriptive statistics of study herds (*n* = 46) are shown in Table 1. Room-level all-in all-out production (AIAO) was utilized in 24 herds (52%) and was not utilized in 22 herds (48%). In control and case herds, AIAO was used in nine (36%) and 15 (71%) herds, respectively. The AIAO production was associated significantly with pleurisy in univariate analysis (*p* = 0.02).

**Table 1:** Basic descriptive statistics of 46 study herds raising finishing pigs in Finland

Variable	*n*	Mean	SD	Median	Min	Max
No. of finishing pigs*						
All herds	46	–	–	730	250	5400
Control herds	25	–	–	500	250	1100
Case herds	21	–	–	1200	300	5400
No. of finishing pig compartments	46	–	–		1	22
No. of finishing pigs/compartment	46	231.3	100.9	–	–	–
No. of finishing pigs/pen	46	10.6	2.8	–	–	–
Piggery constructed (years ago)	41	–	–	14	1	62
Piggery reconstructed (years ago)	13	–	–	7	0	27
No. of caretakers (/1000 finishing pigs)	46	–	–	2.5	0.3	7.3

Mean and standard deviation are reported for normally distributed variables and median, minimum and maximum values for the others

Variables that have a significant association with the main outcome variable (the herd being a case herd with high pleurisy prevalence in slaughterhouse or control herd with low prevalence of pleurisy) are reported separately for control and case herds

*Variables that have a significant association with the main outcome variable (pleurisy) in univariate logistic regression (*p* ≤ 0.05)

Descriptive statistics of clinical signs calculated as continuous variables in study herds are shown in Table 2. In addition to these continuous variables, the proportion of pigs lying on top of each other was handled as a categorical variable: in 27 herds (58.7%), no pigs were lying on top of each other, while in 19 herds (41.3%) at least one pig was lying on top of another.

**Table 2:** Descriptive statistics of clinical signs of finishing pigs after ten weeks of rearing in 46 study herds (continuous variables)

Variable	*n*	Median	Min.	Max.
Coughing, %				
All herds	46	0.1	0	5.5
Control herds	25	0	0	5.5
Case herds	21	0.5	0	1.6
Sneezing, %				
All herds	46	3.4	0	16.0
Control herds	25	3.9	0	15.3
Case herds	21	3.3	1.2	16.0
Tear staining, %				
All herds	44	66.0	1.0	100
Control herds	24	66.9	13	99.0
Case herds	20	56.1	0.95	100
Runts, %				
All herds	46	0	0	40.0
Control herds	25	0	0	40.0
Case herds	21	0	0	1.8
Lameness, %				
All herds	46	1.8	0	8.2
Control herds	25	1.1	0	8.2
Case herds	21	1.9	0	4.6
Acute tail biting, %				
All herds	46	0	0	60
Control herds	25	0	0	3.7
Case herds	21	0	0	60
Healed tail biting, %*				
All herds	45	10.0	0	94.7
Control herds	25	4.3	0	33.7
Case herds	20	13.1	0	94.7
Flank biting, %*				
All herds	46	0	0	50
Control herds	25	0	0	0.99
Case herds	21	0	0	50
Sitting, %				
All herds	45	8.1	0	19.0
Control herds	24	9.0	1	19.0
Case herds	21	5.9	0	17.5
Neurological signs, %				
All herds	45	0	0	0.9
Control herds	25	0	0	0
Case herds	20	0	0	0.9
Conjunctivitis, %				
All herds	46	7.0	0	53.5
Control herds	25	3.3	0	31.6
Case herds	21	12.0	0	53.5
Diarrhoea, %				
All herds	46	0	0	0.06
Control herds	25	0	0	0.04
Case herds	21	0	0	0.04
Skin scratches, %				
All herds	46	0	0	7.5
Control herds	25	0	0	0
Case herds	21	0	0	7.5

* The variables that have a statistically significant association with the main outcome variable (pleurisy) in univariate logistic regression (*p* ≤ 0.05)

The summary of environmental measures in study herds has been collected in Table 3.

**Table 3:** Summary of environmental measures in compartments with finishing pigs after ten weeks of rearing in 46 study herds

Variable	*n*	Mean	SD	Median	Min	Max
Temperature (°C)						
All herds	46	19.4	2.8§	19.0	13	26.5
Control herds	25	19.4	2.8	19.0	13	25.3
Case herds	21	19.6	2.8	19.2	14.7	26.5
Humidity (%)						
All herds	46	67.0	9.4	68.3	43.2	87.4
Control herds	25	67.5	9.0	68.0	43.2	87.8
Case herds	21	68.4	10.0	68.8	51.7	86.8
Airflow (m/s)						
All herds	46	0.2	0.1	0.2	0.1	0.4
Control herds	25	0.2	0.1	0.2	0.1	0.3
Case herds	21	0.2	0.1	0.2	0.1	0.4
Ammonia (ppm)						
All herds	46	5.8	3.4	5.3	1.3	18.3
Control herds	25	5.1	2.5	5.3	1.3	9.0
Case herds	21	6.6	4.2	5.5	1.3	18.3
Temperature of pen wall (°C)						
All herds	43	18.9	3.3	18.9	10.9	25.1
Control herds	24	19.1	2.9	19.0	15.5	24.7
Case herds	19	18.5	3.8	18.9	10.9	25.1
Water flow (l/min)						
All herds	46	1.8	0.8	1.6	0	4.1
Control herds	25	1.7	0.8	1.6	0	4.1
Case herds	21	1.9	0.8	1.6	0.95	3.5
Soiled pens (%)						
All herds	46	21.8	22.4	16.7	0	100
Control herds	25	18.0	18.1	10.3	0	64.3
Case herds	21	26.4	26.4	16.7	0	100
Temperature outdoors (°C)						
All herds	44	4.5	8.9	5.0	−15	25.2
Control herds	24	5.2	9.9	6.9	−15	23.2
Case herds	20	3.6	7.8	2.5	−10	25.2

The summary of meat inspection findings in the study herds after the herd visits is presented in Table 4. The prevalence of pleurisy with the slaughterhouse and herd classification is presented in Table 5.

**Table 4:** Meat inspection findings of the batch slaughtered after the herd visit in 46 study herds

Variable	*n*	Mean	SD	Median	Min	Max
No. of slaughter pigs	46			286.5	58	1530
Mean carcass weight	29	87.0	7.9	87.7	62	106
Condemnations (kg/pig)*						
All herds	27	0.4	0.34	0.3	0.03	1.4
Control herds	15	0.3	0.34	0.2	0.03	1.4
Case herds	12	0.5	0.33	0.3	0.8	1.4
Lean meat (%)	29	60.1	0.9	60.2	57.2	62.1
Whole carcass condemnations (%)*						
All herds	46	0.2	0.3	0.1	0	1.0
Control herds	25	0.1	0.3	0	0	1.0
Case herds	21	0.3	0.3	0.3	0	1.0
Partial carcass condemnations (%)*						
All herds	46	–	–	5.8	1.2	16.2
Control herds	25	–	–	5.0	1.2	16.2
Case herds	21	–	–	7.4	2.2	13.9
Organ condemnations (%)	44	–	–	1.1	0	10.5
Pneumonia (%)	46	–	–	1.3	0	6.2
Pleurisy (%)*						
All herds	46	–	–	5.8	0	60.9
Control herds		–	–	1.9	0	34.5
Case herds		–	–	42.4	2.9	60.9
Milk spots in liver (%)	46	–	–	1.7	0	56.8
Arthritis (%)	46	–	–	2.7	0	10
Abscesses (%)*						
All herds	46	–	–	3.1	0.9	12.7
Control herds	25	–	–	2.7	0.9	11.2
Case herds	21	–	–	4.9	1.4	12.7
Tail biting (%)*						
All herds	46	–	–	0	0	3.6
Control herds	25	–	–	0	0	1.7
Case herds	21	–	–	0.6	0	3.6

Variables that have a significant association with the main outcome variable (pleurisy) are reported separately for control and case herds

Mean and standard deviation are reported for normally distributed variables and median, minimum and maximum values for the others

* Variables that have a significant association with the main outcome variable (pleurisy) in univariate logistic regression (p ≤ 0.05)

**Table 5:** Summary of prevalence of pleurisy in meat inspection of the batch slaughtered after the herd visit in 46 study herds

Variable	*n*	Median	Min	Max
Pleurisy (%)				
All herds	46	5.8	0	60.9
Slaughterhouse 1	14	1.0	0	4.0
Slaughterhouse 2	27	35.9	1.9	60.9
Slaughterhouse 3	5	1.5	0	4.9
Pleurisy (%)*				
Control herds	25	1.9	0	34.5
Case herds	21	42.4	2.9	60.9

* Variables that have a significant association with the main outcome variable (pleurisy) in univariate logistic regression (*p* ≤ 0.05)

Altogether, 690 blood samples from 46 study herds were analysed with two APP serological tests. Of control herds, 25% had high levels of APP2 antibodies. Of case herds, 76.5% had high APP2 antibody levels. Regarding APPIV antibodies, 25% of control and 82% of cases had high levels of APPIV antibodies.

SIV serology was carried out in 40 of 46 study herds for 600 blood samples. Five herds (12.5%), specifically one control and four case herds, had at least one pig with antibodies against SIV.

## Results from statistical modelling

Regarding possible management-related risk factors for a herd being a case herd (i.e. high pleurisy prevalence at slaughter), the herd type “farrow-to-finish” turned out to be a protective factor (OR 0.2, *p* = 008) compared with the herd type “only finishing pigs”. Furthermore, for 500 change in numbers of finishing pigs in a herd, the odds for a herd being a case herd changed by 11.6 (*p* = 0.002). Results of the logistic regression model of for pleurisy are presented in Table 6.

**Table 6:** Results of logistic regression model of risk factors for a herd being a case herd (i.e. a herd with high pleurisy values in meat inspection) of 46 study herds

Variable	OR	SD	*p-value*	95% CI
Farrow-to-finish herd (finishing herd)	0.2	0.3	0.08	0.03–1.2
No. of finishing pigs	1.005	0.002	0.002	1.002–1.008
Constant	0.03	0.03	0.006	0.002–0.4

The reference level for a categorical variable “herd type” is provided in parentheses

## Discussion

The study revealed two factors to be associated with high pleurisy values in pig herds in a country with a low prevalence of many respiratory diseases. A protective effect of “farrow-to-finish” herd type compared with “only finishing pigs” herds emerged regarding odds of having higher pleurisy values in meat inspection (at the 0.1 significance level). In previous studies, contradictory results have been obtained when herd type has been considered in association with pleurisy. Several studies have found that herds that purchase weaners (i.e. finishing pig herds) have a greater risk for respiratory diseases than herds with closed production [2, 15]. On the other hand, farrow-to-finish herds have been observed to have greater odds for having chronic pleurisy in slaughtered pigs [8, 16]. A fairly recent study by Jäger et al. [16] found that if growers are purchased from more than three different farms, the protective effect of finishing herd over farrow-to-finish herd was diminished. In addition to herd type, the AIAO production system has been associated strongly with better respiratory health and, more specifically, lower prevalence of pleurisy [17, 18]. In our study, AIAO production correlated strongly with herd type, and because of this the variable was omitted from multivariate modelling. It is not at all surprising that finishing pig herds were able to empty the piggery at one time and farrow-to-finish herds utilized more continuous flow of animals. In our study, AIAO production was significantly associated with pleurisy in univariate analysis, but surprisingly, case herds utilized more often the AIAO system than control herds. When we investigated only the effect of AIAO production on level of pleurisy and built a statistical model solely for this purpose, our data (results not shown) revealed that herd type acts as confounding variable. When confounding is taken into account, AIAO production seems to have a protective (albeit not significant) effect on pleurisy level.

The present study agrees with the previously described debilitating effect of growing herd size on prevalence of pleurisy [6, 10, 11, 19]. This effect could be related to infection pressure because bigger herds are more likely to need to purchase more animals, which is accompanied by an increased risk of introducing pathogens or naïve animals into the herd. Disease dynamics (i.e. better possibilities for spread and maintenance of airborne infection) also differ between bigger and smaller herds [18]. Considering environmental risks, it might also be more difficult for bigger herds to control optimal air quality, especially if the compartments are large. However, this was not the case in our study, where room size did not differ between large and small herds.

Herd type and size are commonly and inextricably associated with specific types of management practices, e.g. farrow-to-finish herds do not buy weaners or growers and finishing herds more commonly are able to practice AIAO production. However, the herd type itself, when used in the sense of sourcing animals, seems to be too vague a definition for choices made on a certain farm regarding infection pressure. To be able to reliably predict risk factors for chronic pleurisy, these management practices need to be defined in more detail. Earlier studies have been able to find several risk factors for pleurisy in the slaughterhouse such as number of pigs per pen [2], pig density in the neighbourhood [3, 4], low health status of the herd [3], poor biosecurity [4], lack of disinfection of the farrowing room [11], no cleaning and disinfection [16], lack of complete AIAO production [3, 16, 19], mixing of pigs [3, 16], season [3, 4, 20], mean temperature below 23 °C in the finishing unit [11], low weaning age [2], low airspace [4] and higher particulate matter concentration [21]. Jäger et al. [16] observed that keeping pigs with more than a one-month age difference in the same airspace acted as a risk factor for pleurisy. Cleveland-Nielsen et al. [3] found that feeding only dry feed protected the herd from high pleurisy values. However, some studies have not observed any association between non-infectious risk factors studied and pleurisy in the slaughterhouse [1, 22]. No effect of detailed management factors, clinical signs or environmental measurements on herd-level prevalence of pleurisy was found on present study. This could at least party be caused by a lack of statistical power as discussed later in this text. Regarding clinical signs and environmental measurements, only a single assessment of these factors was carried out during the study, which hardly can be considered as a representative estimation of these factors for the whole rearing period of finishing pigs. Furthermore, post mortem inspection results (including pleurisy) were acquired from more numerous finishing pigs than clinically examined ones. These uncontrolled factors may have caused bias in results and hence a possible association was not observed. Some of the significant associations between pleurisy and management or health factors have been found only in univariate analyses, while in multivariable analysis the associations have disappeared. This highlights the need to be able to model several correlated variables simultaneously. Hurnik et al. [17, 23] attempted to overcome these kinds of problems typical for surveys by using a factor analysis. Regarding pleurisy, they found only one common observed feature of herds having greater prevalence of pleurisy: extensive type of farming. However, there is convincing evidence in the literature, as summarized above, that management choices related to infection pressure have a considerable effect on prevalence of chronic pleurisy. This is further supported by the observation of the protective effect of farrow-to-finish herd type and small herd size in our study.

We did not find any association between serological results and pleurisy prevalence measured in post mortem meat inspection. Controversial results have been previously reported regarding serology and pleurisy registered in meat inspection [2, 8, 13, 24]. In case of endemic disease, serology might not be the most useful tool in diagnosis or at least paired samples are needed [25]. Also, the timing of the infection remains unknown in our study, as we are dealing with endemic disease. For example, Wallgren et al. [6] found different serological patterns during the growing time of pigs in four herds. They showed that repetitive sampling helped to pinpoint the actual causative agent and disease pattern in different herds.

In our study, only five herds had antibodies against SIV. Even though four out of these five herds were case herds and only one a control herd, the total number of SIV-positive herds was too small for statistical modelling. SIV, both H1N1 and A(H1N1)pdm09, was first found in Finland in 2008 and 2009 [26], and both of the strains have thereafter spread throughout the country. The arrival of this new pathogen to a previously naïve population may have played a role in the increased pleurisy prevalence observed in meat inspection during recent years. However, based on current results, it remains unclear whether SIV plays a role in chronic pleurisy in Finland.

In this study, herds were defined as cases or controls based on detection of pleurisy for a longer time period than only one batch. Furthermore, we gathered management-related data during herd visits, which enabled us also to inspect the pigs clinically. These choices should have made both the allocation of herds into cases or controls and the collection of data reliable. The results should be more valid than in studies where allocation has been done based on a single batch of slaughtered pigs or where the data have been collected with questionnaires sent to farmers or telephone interviews [3, 17, 20]. Purposive sampling was utilized in our study to highlight the differences between case and control herds. Typically, this type of sampling leads to valid estimates if otherwise conducted properly, but might restrict extrapolation of study results. Study herds had on average 1000 finishing pigs, which is approximately double the typical Finnish pig herd size. However, 50% of finishing pigs in Finland are produced in herds having more than 1000 finishing pigs. While descriptive statistics may not be representative of all Finnish pig herds, the results may well be applied in modern pig production. As always in a case-control study, the role of cause and consequence cannot be proven.

Almost 70% of herd owners asked to participate in the study were willing to be involved. As case herds were overrepresented amongst opt-out herds, there is a possibility of selection bias in the data. The most frequent reason for opting out was “not willing to participate”. Almost 60% of these herds were case herds and the reason for refusal was frequently that “participation will not help them to overcome the high pleurisy problem that they have”. The majority of opt-out control herds no longer had pigs, and this was the second most common reason for opting out. Generally, opt-out herds had a larger herd size than opt-in herds. Therefore, it is possible that the herds not willing to participate in the study might have been not only larger, but also not as keen to take active measurements to improve their management. This kind of perceived attitude may have some (possibly negative) influence on overall management of herds. Possible selection bias may have caused study herds to resemble each other more in terms of influential management variables than they actually do in the source population, leading to an underestimation of the severity of risk factors.

Lack of statistical power might have caused several factors observed to differ between case and control herds to be seen as statistically non-significant. For example, for the present sample size (at least 24 herds in both groups) the presumed difference in exposed and non-exposed herds should have been 40 and 80% in most influential risk factor (herd type). The realized distribution (36% vs. 80%) fulfilled that criteria well and statistical significance was seen. However, as calculated for another categorical variable (NH3 concentration), the observed proportions of exposed and non-exposed herds were 61 and 52%, respectively, which may lead in a lack of statistical power. Furthermore, quite many possible risk factors were included in the initial multivariable model, which probably exceeded the power of the current study.

## Conclusions

Finishing herd type and herd size were observed to act as risk factors for a herd to be a case herd (i.e. a herd having high prevalence for chronic pleurisy detected in meat inspection). No clinical signs of the pigs, management-related factors or environmental measurements were associated with pleurisy values. As previously known, in endemic and subclinical infections such as APP, herd factors are important, but detailed risk factors seem to be difficult to identify.

## Data Availability

The datasets supporting the conclusions of this article are available in the Open Science Framework repository, https://osf.io/usmy7/.
